# Improving performances of the emergency department using discrete event simulation, DEA and the MADM methods

**DOI:** 10.1177/2055207616664619

**Published:** 2016-08-19

**Authors:** Alireza Gharahighehi, Amir Saman Kheirkhah, Ali Bagheri, Ehsan Rashidi

**Affiliations:** 1 Department of Industrial Engineering, Faculty of Engineering, Bu-Ali Sina University, Iran; 2 Department of Industrial Engineering, Sharif University of Technology, Iran; 3 Department of Industrial Engineering, Islamic Azad University, South Branch, Iran

**Keywords:** Emergency department, discrete event simulation, non-homogenous arrivals, specimen queues, DEA, extended VIKOR method

## Abstract

This article presents a method by which performances at an emergency department (ED) in a large hospital in Iran could be improved, where the long waiting times and unbalanced utilization create problems for patients and ED staff. This method firstly simulates patient flow in the ED and finds bottlenecks that cause inefficiency in ED performance. In the simulation model, patient arrival is assumed to be non-homogenous and the operation of medical tests such as MRI, CT scan, pathology testing, laboratory testing, ultrasonography, and radiology are detailed and virtual queues of patients' specimens are considered separately from patient queues. Based on the simulation reports of the current situation and target criteria, what-if scenarios were used to design scenarios that could improve ED performance. This method used the data envelopment method (DEA) to determine efficient scenarios, analytic hierarchy process (AHP) to specify the weight of each criterion, the Delphi method to specify suitable utilization rates for various resources, and the extended Vlsekriterijumska Optimizacija I KOmpromisno Resenje (VIKOR) method to compare data on 95% confidence intervals from efficient scenarios and to rank scenarios by considering conflicting criteria. Implementing the first scenario in the ranking would reduce acute patients' overall waiting time by approximately 5%, and it doesn't require any additional investments.

## 1. Introduction

Health services represent a considerable part of the economic, social, and political aspects of a country; for instance, health care accounts for more than 16% of the gross domestic product (GDP) in the United States.^1^ That is why researchers in various fields work on improving the efficiency and quality of health services to satisfy patients and the health community.

The emergency department (ED) is one of the most important and perhaps the most significant part of a hospital; it plays a central role in furthering the objectives of a hospital to improve the quality of its services. The ED is a place that provides health services 24 hours a day, seven days a week to injured and sick patients, and medical tests are conducted there until patients' health conditions are stabilized.^2^ On the one hand, patients from different hospitals and medical centers are moved to the ED for their vital signs to be stabilized before they are admitted to health centers, and on the other hand, a considerable number of outpatients also directly have recourse to an ED. This part of the hospital is often faced with acute patients who have recourse to it without appointment. Because of the uncertainty and the acuity of patient conditions, making the right managerial decisions is more complicated than for patients from other departments. Patients who have recourse to an ED usually have unstable and life-threatening conditions; this means that any delay may contribute to patient death. Delay can occur at an initial point of the chain, such as when calling the ambulance centers, or it can occur in emergency centers and hospitals. For example, the shortage of hospital beds, physicians, nurses, ED staff (e.g. admissions, services), lack of medicines and consumables, and patient flow may cause delay. Because of the high value attached to health, it is necessary to reduce delays and improve the regulation of patient flow.

Many indicators are used to indicate an ED's performance; for example, the average length of stay (LOS), waiting time, number of rejected patient(s) because of unavailability of beds, and patient throughput. LOS is the total time (in hours) a patient spends in the ED from the time of registration/triage to when the patient leaves the ED. Waiting time is the time a patient must wait because the server is busy.

Undoubtedly the biggest problem for most EDs is overcrowding caused by patients who need care. ED overcrowding can be described in different ways: an excessive number of patients in the emergency department, patient examinations in the corridor,^3^ long waiting times, the diversion of ambulances, and departure from the ED without receiving health services. The consequences of this crowding are patient rejection, disruption of ED operations, service quality deterioration, reduction of staff and patient vitality, increasing stress among emergency staff, patient dissatisfaction, reduction of physicians' productivity, and adverse effects on the educational performance of ED interns.^4^

Many solutions are proposed for reducing overcrowding in the ED such as easing access to clinics, increasing emergency beds and space, increasing the capacity for radiology, laboratory, and other medical tests, and reducing patient entry to congested EDs. To overcome and control this problem, ED managers are expected to have good knowledge of what is happening in the ED. This means that managers should be aware of staff and facility utilization, the average waiting time for each patient in every part of the ED, and the factors leading to bottlenecks that cause inefficiency. Once the current situation is understood, what-if scenarios can be used to establish ways of making decisions that result in ED performance improvement. Since the ED is a complicated system, analytical methods such as queuing theory can model a small portion of it. Because of the complexity of the ED system, simulation is a useful tool to model it.

The main objective of this research is improving performances of ED using simulation, the data envelopment method (DEA), and the Delphi and Multi Attribute Decision Making (MADM) method. In order to reduce waiting time and balance utilization, some changes should be made. Thus these changes have initially been identified and prioritized. In this paper we aim to improve the performances of the intended ED by capacity planning and revising procedures. Procedures means not the medical but systematic procedures. So we tried to improve the ED's performances by existing treatment processes.

There are some contributions in this research. First, as patient arrival rates are not constant during the hours in a day and days in a week, patient arrival was considered to be non-homogenous in terms of the day and week simultaneously. Second, two kinds of queues were analyzed: patient queues and specimen queues. Analyzing the specimens flow would help us to identify problems in the medical test process. Third, DEA and MADM methods were applied together to specify efficient scenarios and rank them. Fourth, because of the uncertain environment of the simulation model, instead of average numbers, 95% confidence interval (CI) data were used and therefore an extended form of Vlsekriterijumska Optimizacija I KOmpromisno Resenje (VIKOR) was applied that could rank scenarios with interval data. All in all, these premises made the model a new approach in order to reflect the reality as much as possible in the model and also aimed to assist decision makers to select the best alternative by a more effective tool.

## 2. Literature review

Many researchers have already made good use of discrete event simulation (DES) to model patient flow. They have used simulation to find obstacles, improve productivity, and identify problems that cause long waiting times or patient dissatisfaction. Simulations have been widely used in health care systems in hospitals, clinics, and small health centers. Jacobson et al.^5^ provided an overview of the application of simulation in health care systems including hospitals, clinics, EDs and pharmacies. Paul et al.^6^ reviewed simulation studies with respect to five aspects: goals, modeling, data collection, patient flows and study results. Gul and Guneri^7^ provided a comprehensive review on applications of simulation in the ED. In their review, the studies are classified into four main groups. The first group includes studies using DES. The second one comprises studies using DES and other methods such as heuristics, six sigma and queuing theory. Studies using agent-based simulation (ABS) are introduced next, and the last group uses ABS and other methods. They showed that DES is the preferred modeling methodology among others.

Research in this area can be categorized into two groups: first, simulation has been used for capacity planning and identifying ways and scenarios for improving ED performance by changing capacity levels; second, research has focused on the processes and procedures of the ED. A greater proportion of research falls into the first group, as the scenarios are more operational.

The space and capacity of the ED is mostly dependent on beds and rooms. The main reason for the bustle and long waiting time in the ED is the unavailability of beds. Takakuwa and Shiozaki^8^ showed that waiting for access to emergency beds accounts for 59% of waiting time. Komashie and Mousavi^9^ examined two scenarios concerning emergency beds. In the first scenario they added one bed for acute patients and in the second scenario they added six beds for the entire ED. Interestingly, it was observed that the second scenario was only slightly better than the first one for patient waiting times despite the fact that in the second one the increase in capacity was six times more than the first one. Samaha et al.^10^ came to the conclusion that increasing the number of beds or ED space would not have a significant effect on LOS. Kirtland et al.^11^ observed that if a patient who is waiting for a bed is being kept in a therapeutic area instead of a waiting room, the patient's waiting time would be on average reduced by 14.1 minutes.

There is other research about capacity planning that is not on beds; human resources and equipment have also been studied. Rossetti et al.^12^ simulated 18 schedule plans for ED staff and among these they identified an option that reduced LOS by 14.5 minutes. They also showed that the implementation of this option would increase physicians' utilization and would reduce long waiting times. Tan et al.^13^ realized that physicians are capacity bottlenecks and by increasing their capacity, LOS will be decreased. Some studies have looked at the equipment and facilities as factors in improving ED performance.

Changing and replacing the processes and procedures of ED is the other way of overcoming long waiting times. Samaha et al.^10^ showed that the addition of fast-tracking resulted in a considerable decrease in LOS. Fast-tracking is an approach that sees routine patients in a dedicated “fast track” area, which is staffed by a dedicated nurse practitioner.^10^ Kirtland et al.^11^ also concluded that applying this approach will save 15.5 minutes for the patient. Some studies have concentrated on standards and protocols. Kirtland et al.^11^ pointed out that changing triage protocols and allowing triage nurses to give specific medical tests like radiology decreased LOS. Ashour and Okudan Kremer^14^ introduced an interactive algorithm for the triage process using the fuzzy analytic hierarchy process (FAHP) and the multi-attribute utility theory (MAUT), which improved performance.

In making a comprehensive decision, the analyzer should take into account a combination of mentioned scenarios. Eskandari et al.^15^ used the MADM method to choose the best scenario, which included increasing the number of computed tomography (CT) scan machines and changing patient priority for magnetic resonance imaging (MRI). Santibáñez et al.^16^ considered three factors in the design of scenarios: operational factors, appointment scheduling factors, and resource allocation, which includes both capacity planning and process changing. Al-Refaie et al.^17^ used simulation and DEA in order to reduce waiting time. Their results showed that the best scenario depends on workload sharing assignments, reducing patient's average waiting time from 195 to 183 minutes, increasing the number of patients served from 8853 to 8934 patients, and improving the nurses' utilization from 52% to 62%.

In the health care scope, the decision-making process happens frequently. In this process, the manager should decide based on some criteria that sometimes conflict. In this situation MADM is a useful tool that can help the manager to find the best decision. Thokala and Duenas^18^ provided a review on the applications of these methods in health care and performed a comparison between these methods and applied them to a hypothetical case study, and outlined potential strengths and weaknesses of these methods. Nobre et al.^19^ used the MADM approach to support public health decision making considering the fuzziness of the decision goals. They applied a method known as Interactive and Multi criteria Decision Making (TODIM) to evaluate alternatives. Jlassi et al.^20^ showed that in order to improve performance, it is necessary to add a specialist physician or an informed general physician. To choose which physician to add, the researchers used the fuzzy Preference Ranking Organization Method for Enrichment Evaluations (PROMETHEE) method. Gul et al.^21^ used an integration of simulation and interval type-2 fuzzy AHP (IT2FAHP), and ELimination Et Choix Traduisant la REalité (ELECTRE) for an ED in order to evaluate different scenarios and select the best scenario considering different numbers of staff.

## 3. System description

The ED of a government hospital in Iran was analyzed and modeled in this study. This ED is one of the biggest and busiest in Iran; it has 36 inpatient beds and also 16 beds for stabilizing the patients' conditions. The ED has a laboratory and radiology, but for other medical tests such as MRI, ultrasonography, pathology, and CT scans patients should be referred to the hospital. There were 22 nurses and three physicians on the first shift and 15 nurses and three physicians on the second shift.

**Figure 1.**
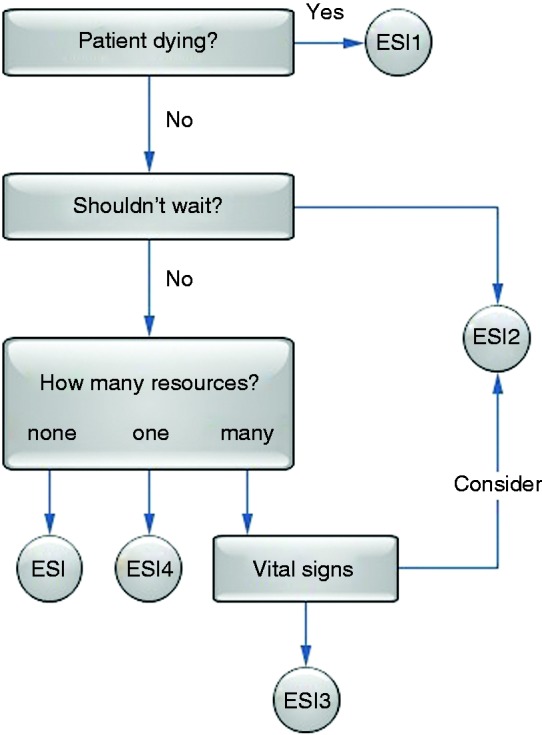
Emergency Severity Index conceptual algorithm.^15^

When a patient arrives, the first step involves the triage nurse. Based on the level of acuity of the patient's condition, the triage nurse specifies a flow for the particular patient. The current acuity level system at the ED is the Emergency Severity Index (ESI) system. This system is a five-level measure to rate patient acuity, from ESI1 (most urgent) to ESI5 (least urgent). This system categorizes ED patients both by acuity and resource needs.^22^ Figure 1 illustrates how the triage nurse categorizes the patient using this five-level tool.

**Figure 2.**
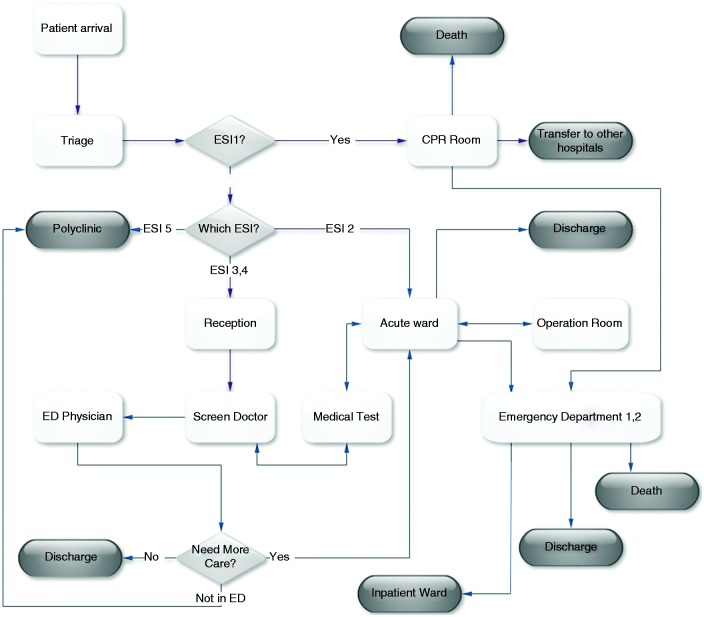
Conceptual model of patient flow in the emergency department (ED). ESI: Emergency Severity Index; CPR: cardiopulmonary resuscitation.

After specification of the ESI level by the triage nurse, the patient has recourse to the next stage of ED treatment. The patient's flow is completely based on his or her ESI level. ESI1 patients are transferred to the cardiopulmonary resuscitation (CPR) room and if recovery operation is successful, they are transferred to the inpatient wards. ESI2 patients are transferred to the acute part until they reach a stable state. ESI3 and ESI4 patients are examined by a screening doctor, who determines the required medical tests for the patients. After understanding the current patient flows, we designed a conceptual model to explain the patient flows in the ED schematically, which helped us to build our simulation model. Figure 2 shows the conceptual model for the ED. According to Figure 2, the severity levels will determine the patient flows. In addition, the triage nurse and the screening doctor have key roles in determining which patients should be referred to which part of the ED or hospital. The flow starts when a patient arrives and finishes when a patient is discharged, has died, or is admitted to other parts of the hospital.

## 4. Methodology

For this study eight steps were undertaken to improve the ED's performance indicators such as waiting time, utilization and leave with own responsibility (LWOR). These steps are as follows:

– Data collection.
– Modeling the system and simulating it.
– Validating and verifying the model.
– Determining the criteria that are important in the manager's mind. They are also performance indicators.
– Determining the weight of each criterion.
– Designing scenarios based on critical bottlenecks.
– Finding efficient scenarios.
– Ranking the efficient scenarios.

### 4.1. Data collection

The data collection was conducted in two ways: first, data were gathered from the hospital's health information system (HIS) and, second, information was obtained from interviews with ED staff, including nurses. We extracted useful information about patient arrivals and service times from the HIS; this information covered approximately two years from Saturday, April 23, 2011, to Friday, April 12, 2013. From these data we calculated the non-homogenous patient arrival rate and their probability distribution, and also the service time distribution for many parts of the ED. We used Input Analyzer by Arena® simulation software to specify the distributions that we needed for our model.

### 4.2. Simulation

When we gathered data and found the required distributions, they were inserted into our simulation model. We considered some special features in our simulation model as follows: non-homogenous patient arrival, virtual specimen queues, and queues that the ED shares with other parts of the hospital to access medical test resources.

#### 4.2.1. Non-homogenous patient arrival

**Figure 3.**
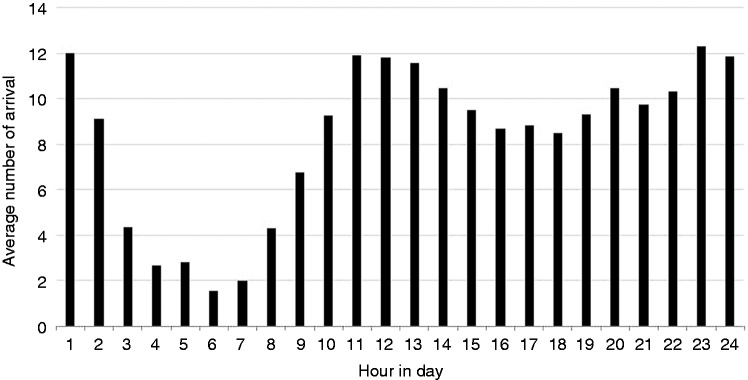
Average number of arrivals every hour of one day.

Usually patient arrival does not take a homogenous form. The variability of patient arrival time can be defined according to time scales that vary according to the hour, day, week, month, or even season. For instance, the average patient arrival time in winter is significantly different from arrival in summer. In our study we assumed that patient arrival would be non-homogenous in terms of day of the week and time of day, since we knew that there would be significant differences in patient arrivals in the mornings, evenings and nights. Figure 3 illustrates the average number of arrivals at every hour of the day.

**Figure 4.**
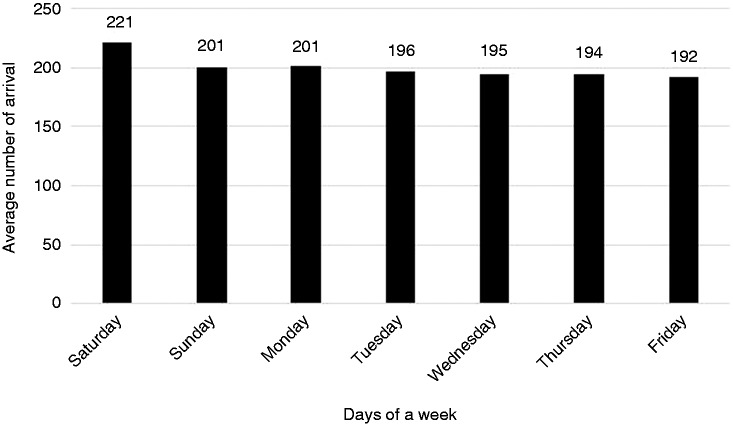
Average number of arrivals every day of a week.

As Figure 3 shows, the busiest time period in the day was from 10:00 p.m. to 11:00 p.m.; on the other hand, the ED seemed to be relatively free from midnight onwards, especially from 5:00 a.m. to 6:00 a.m. Figure 4 illustrates the average number of arrivals on every day of the week. As Figure 4 shows, the busiest day of the week was Saturday, while Friday was uncrowded compared to other days.

We inserted the arrival data that we had obtained from the hospital's HIS into Input Analyzer and determined the patient arrival probability distribution for each time interval. These distributions were entered in Arena®.

#### 4.2.2. Specimen virtual queues

**Figure 5.**
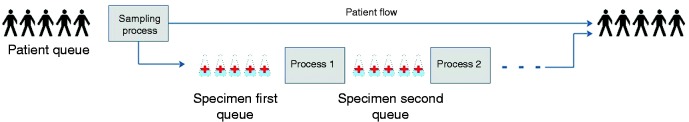
Patient queue and specimen queues.

Most relevant studies have not considered specimen queues. When a patient finishes a test such as a laboratory test, the rest of the patient flow is paused until the test results are prepared. Therefore, in this system we can consider two kinds of queue: the first is the patient queue; the second is the specimen queue. By considering these queues when we ran our model, we could make more accurate decisions based on final reports. We were able to choose different priorities for each of these two kinds of queue in the ED, as depicted in Figure 5.

In the simulation model we considered the queues in MRI, CT scanning, ultrasonography, laboratory and pathology testing.

#### 4.2.3. Shared use of medical test facilities

In our case facilities such as ultrasonography, MRI, CT scan, and pathology were used as pooled resources. So when we wanted to make decisions about them, we could consider sharing queues according to different priorities and severities. In our model based on scenarios we could adopt different priorities on both patient and specimen queues. These priorities are not just for ED patients but also other patients from other parts of the hospital.

### 4.3. Model verification and validation

Model verification means the model acted as was intended by the model designer.^23^ There are various ways to verify simulation models. One of them involves changing the model parameters and observing whether the model behaves as is intended; for instance, we increased the patient arrival rate and some of the service times and observed the results. For this purpose animation of simulation can be used to ensure that what happens in the animation is exactly the intended behavior that was defined in the conceptual model. We decreased the animation speed factor to follow patients and specimen flows at all levels. This assured us that the model acted as intended.

Model validation examines how realistic the model is, or in other words how the model reflects real conditions.^23^ Of course, models cannot exactly reflect the real system; at best they are a good approximation of it. So, to validate our model we had to observe how similar the simulation model was to the real system. The best way is to compare simulation outputs and real data from the HIS and ensure that the real data fall within the 95% CIs of the simulation outputs. Accordingly, we compared the LOS of three levels of patients in the simulation outputs with real data. Real data, which were exported from HIS, had to be adjusted because the numbers did not contain the waiting times for triage and for patients who had died in the ED. After adjusting waiting times, the time intervals included real data and our model was validated. The results are shown in Table 1.

**Table 1.** Validation results.

	LOS (min)	95% confidence intervals for LOS
ESI2	915	(745.13–946.67)
ESI3	3290	(3214.40–3352.59)
ESI4	4857	(3214.40–5201.88)

LOS: length of stay.

### 4.4. Designing criteria

From the hospital's point of view there were four criteria that should be mentioned in decision making, which are as follows:

- Waiting time

This factor reflects the average time that a patient waits to receive all the required services. This was definitely a negative factor and we wanted to decrease the waiting time. Waiting times for four ESI levels were considered in the decision matrix. The patients considered as ESI5 did not enter our matrix because their flow in the ED finished after triaging, when they had recourse to the polyclinic. The average waiting times for ESI1 to ESI4 are 22, 141, 1028 and 1829 minutes.

- Utilization

This criterion could be either negative or positive. It is not correct to assume that 1 was the ideal point of utilization; the utilization level of 1 results in an increased waiting time and patient dissatisfaction. So the trade-off point should be specified, balancing the idleness of resources and waiting times. According to the Delphi method, which was conducted by doctors, hospital managers and professionals, the optimum rate of personal utilization and tools utilization were determined to be 0.7 and 0.75, respectively.

- Cost

This factor reflects the cost of implementing the scenarios that were defined in our decision matrix. This cost has to do with increasing the capacity of physicians, nurses, staff and equipment.

- LWOR

LWOR patients wait a long time for medical services and are deterred from continuing, so leave the ED, taking responsibility for this action themselves.

### 4.5. Determining weight of each criterion

When a decision maker wants to rank alternatives based on criteria, the weight of each criterion should be determined on the basis of its importance and the decision maker's point of view. In this study the analytic hierarchy process (AHP) was used to specify the relative importance of each criterion.^24^ To specify the weight of each criterion a questionnaire was designed. It contained a pair-wise comparison on the basis of nine scales of the AHP method. The ED manager filled out a questionnaire and the pair-wise comparison matrix was concluded. The weights of the criteria can be determined by using the greatest eigenvalue of the matrix and the corresponding eigenvector. Consistency of the decision maker should be examined. In this study consistency ratio (CR) was calculated and it was less than 0.1 (CR = 0.006 < 0.1) so it indicates satisfactory consistency. Table 2 shows the numbers that are used in pair-wise comparison. Based on Table 3, which represents a pair-wise comparison matrix, weights of the criteria were calculated. Table 4 shows these weights.

**Table 2.** The fundamental of absolute numbers.

Definition	Intensity of importance
Equal importance	**1**
Weak importance	**2**
Moderate importance	**3**
Moderate plus importance	**4**
Strong importance	**5**
Strong plus importance	**6**
Demonstrated importance	**7**
Very, very strong importance	**8**
Extreme importance	**9**

**Table 3.** Comparison matrix.

	Waiting time				Utilization												LWOR	Cost
	ESI1	ESI2	ESI3	ESI4	Acute	Beds	CT	MRI	CT radiologist	MRI radiologist	MRI reception	MRI typist	Pathologist	Screen doctor	Sonography radiologist	Triage		
ESI1	1	–	–	–	–	–	–	–	–	–	–	–	–	–	–	–	–	–
ESI2	1	1	–	–	–	–	–	–	–	–	–	–	–	–	–	–	–	–
ESI3	3	2	1	–	–	–	–	–	–	–	–	–	–	–	–	–	–	–
ESI4	4	3	2	1	–	–	–	–	–	–	–	–	–	–	–	–	–	–
Acute	3	2	2	1	1	–	–	–	–	–	–	–	–	–	–	–	–	–
Beds	3	3	1	1/2	1	1	–	–	–	–	–	–	–	–	–	–	–	–
CT	3	4	2	1	1	2	1	–	–	–	–	–	–	–	–	–	–	–
MRI	4	2	1	1	1	2	1	1	–	–	–	–	–	–	–	–	–	–
CT radiologist	7	5	3	2	2	3	2	2	1	–	–	–	–	–	–	–	–	–
MRI radiologist	7	5	3	2	2	3	2	2	1	1	–	–	–	–	–	–	–	–
MRI reception	9	5	4	3	3	4	2	3	1	1	1	–	–	–	–	–	–	–
MRI typist	9	6	4	3	3	4	2	3	1	1	1	1	–	–	–	–	–	–
Pathologist	8	5	3	2	2	3	2	2	1	1	1/2	1/2	1	–	–	–	–	–
Screen doctor	7	3	2	2	2	2	1	1	1/2	1/2	1/2	1/2	1/2	1	–	–	–	–
Sonography radiologist	8	5	3	2	2	3	2	2	1	1	1	1/2	1	2	1	–	–	–
Triage	7	3	2	2	2	2	1	1	1	1	1/2	1/2	1/2	1	1/2	1	–	–
LWOR	1/2	1/3	1/4	1/5	1/4	1/3	1/6	1/5	1/9	1/9	1/9	1/9	1/9	1/9	1/9	1/9	1	–
Cost	2	1/2	1/2	1/3	1/2	1/2	1/3	1/2	1/5	1/5	1/8	1/7	1/5	1/4	1/4	1/3	2	1

ESI: Emergency Severity Index; CT: computed tomography; MRI: magnetic resonance imaging; LWOR: leave with own responsibility.

**Table 4.** Weight of each criterion.

Criteria		Weight	
Waiting time	ESI1	0.15	0.35
	ESI2	0.1	
	ESI3	0.06	
	ESI4	0.04	
Utilization	Acute	0.045	0.3
	Beds	0.06	
	CT	0.035	
	MRI	0.04	
	CT radiologist	0.02	
	MRI radiologist	0.02	
	MRI reception	0.015	
	MRI typist	0.015	
	Pathologist	0.02	
	Screen doctor	0.03	
	Sonography radiologist	0.02	
	Triage	0.03	
LWOR		0.2	0.2
Cost		0.1	0.1

ESI: Emergency Severity Index; CT: computed tomography; MRI: magnetic resonance imaging; LWOR: leave with own responsibility.

### 4.6. Determining scenarios

Based on the simulation reports, we established 10 scenarios that could improve ED performance, as shown in Table 5. They focus on bottlenecks in the system and are designed at the operational level of decision making.

**Table 5.** Proposed scenarios.

	Scenario description
Scenario 1	Current situation (without any change in the system)
Scenario 2	Increasing four beds in free space of ED to increase inpatient ward
Scenario 3	Employing one shift triage nurse from 12 a.m. to 12 p.m.
Scenario 4	Increasing working time of CT scan radiologist from four hours to eight hours
Scenario 5	Employing one shift GP from 12 a.m. to 12 p.m.
Scenario 6	Assigning priority based on severity of patient in every patient queue
Scenario 7	Increasing one eight-hour shift MRI reception
Scenario 8	Increasing one eight-hours shift MRI reception, MRI radiologist and MRI typist
Scenario 9	Increasing one four-hour shift for radiologist and typist in ultrasonography
Scenario 10	Employing one pathologist to increase pathology capacity

ED: emergency department; CT: computed tomography; GP: general practitioner; MRI: magnetic resonance imaging;

### 4.7. Finding efficient scenarios

When the scenarios were established, DEA was used in order to find efficient scenarios. DEA is an approach based on a linear programming (LP) model for evaluating comparative efficiencies of decision-making units (DMUs) with usual inputs and outputs. It is used for placing and analysis of DMUs such as industries, universities, hospitals, cities, facilities layouts, and etc.^25^

We ran the model in DEA software based on 10 scenarios with one input and three outputs, which are presented in Table 6. In Table 6, DMUs are scenarios, the input is the average number of patient arrivals in a day, the first output is the number of LWOR patients, the second output is average waiting time and the last one is cost. All outputs are normalized. Our model is output oriented, envelopment model and hybrid. As presented in Table 7, four scenarios are efficient because their scores are 1 (maximum efficiency); six other scenarios are inefficient and needed to be replaced. In each row by defining benchmark lambda, we indicate that an inefficient scenario can be replaced. For instance, scenario number 7 can be used instead of number 1, and scenario number 2 can be replaced by 6, 7, and 10.

**Table 6.** The input and outputs of DEA model.

DMU	Input 1	Output 1	Output 2	Output 3
S1	200	0.110272	0.052931	0
S2	200	0.085037	0.040818	0.666667
S3	200	0.063293	0.030381	0.4
S4	200	0.083902	0.040273	0.2
S5	200	0.084785	0.040697	0.533333
S6	200	0.103471	0.049666	0.999999
S7	200	0.243029	0.116654	0.2
S8	200	0.081465	0.039103	0.999999
S9	200	0.085861	0.041213	0.4
S10	200	0.085542	0.4106	0.533333

DEA: data envelopment method; DMU: decision-making method.

**Table 7.** DEA results.

No	DMU	Score	Benchmark (lambda)
1	S1	0.45374	G (1.000000)
2	S2	0.724441	F (0.900313); G (0.099686); J (0.000001)
3	S3	0.478817	F (0.794240); G (0.205758); J (0.000002)
4	S4	0.427433	F (0.334887); G (0.665112); J (0.000000)
5	S5	0.639842	F (0.791925); G (0.208074); J (0.000001)
6	S6	1	F (1.000000)
7	S7	1	G (1.000000)
8	S8	1	F (1.000000)
9	S9	0.560021	F (0.642825); G (0.357175)
10	S10	1	J (1.000000)

DEA: data envelopment method; DMU: decision-making method.

### 4.8. Ranking scenarios

After specifying the criteria and the scenarios and finding efficient scenarios, we ranked them based on the criteria. In this study we used the VIKOR method. This method focuses on ranking alternatives by considering conflicting criteria. It uses the ideal solution and ranks alternatives based on their closeness to the ideal solution. It has some advantages. First, it has an extended form regarding interval data. Second, it is a suitable method when criteria are conflicting and non-commensurable. And finally, it employs ideal solutions in its steps.

Because of the uncertain environment of our model, we used 95% CI data instead of average numbers. For this study we could not use the simple form of VIKOR; therefore, the extended form of VIKOR was used as this is suitable for ranking alternatives regarding interval data. According to Sayadi et al.,^26^ this method uses the positive ideal solution (PIS) and the negative ideal solution (NIS) to rank scenarios.

Working steps of the VIKOR method are as follows:

- Normalizing values: In this step the rating of jth criterion for ith scenario, denoted as x_ij_, should be normalized. Normalized values are calculated using formula (1)

**Figure:**
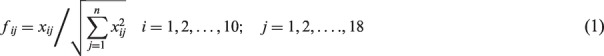
- Finding NIS and PIS: These values show the worst and the best values in every criterion. They can be calculated from formula (2) and (3)
  (2) $$f_{j}^{*}=\max_{i}\{f_{\mathrm{ij}}^{U}|jɛI\}\;\text{or}\;\min_{i}\{f_{\mathrm{ij}}^{L}|jɛJ\}\;i=1,2,\ldots ,10;\;j=1,2,\ldots .,18$$
  (3) $$f_{j}^{-}=\min_{i}\{f_{\mathrm{ij}}^{L}|jɛI\}\;\text{or}\;\max_{i}\{f_{\mathrm{ij}}^{U}|jɛJ\}\;i=1,2,\ldots ,10;\;j=1,2,\ldots .,18$$

Where *I* is associated with positive criteria and *J* is associated with negative criteria. Waiting times, LWOR and cost are negative criteria. As mentioned about utilization, it is a two-fold criterion. So if the utilization value is lower than the trade-off point, it will be considered as a positive criterion, and if the value is more than the trade-off point, it will be considered as a negative criterion.

(c) Calculating the distances of scenarios to the ideal solution: Ideal solutions are needed in calculating $S_{i}=(S_{i}^{L}S_{i}^{U})$ and $R_{i}=(R_{i}^{L}R_{i}^{U})$. The solution obtained by min *S*_*i*_ is with a maximum group utility (“majority” rule), and the solution obtained by min *R*_*i*_ is with a minimum individual regret of the “opponent.” In fact *S*_*i*_ and *R*_*i*_ represents the average and the maximum gap from ideal solution. According to Sayadi et al.,^26^ *S*_*i*_ and *R*_*i*_ can be calculated by formulas (4), (5), (6) and (7).

**Figure:**
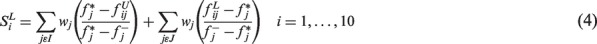

**Figure:**
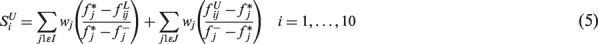

  (6) $$R_{i}^{L}=\max\{w_{j}(\frac{f_{j}^{*}-f_{\mathrm{ij}}^{U}}{f_{j}^{*}-f_{j}^{-}})|jɛI,w_{j}(\frac{f_{\mathrm{ij}}^{L}-f_{j}^{*}}{f_{j}^{-}-f_{j}^{*}})|jɛJ\}\;i=1,\ldots ,10$$
  (7) $$R_{i}^{U}=\max\{w_{j}(\frac{f^{*}-f_{\mathrm{ij}}^{U}}{f_{j}^{*}-f_{j}^{-}})|jɛI,w_{j}(\frac{f_{\mathrm{ij}}^{L}-f^{*}}{f_{j}^{-}-f_{j}^{*}})|jɛJ\}\;i=1,\ldots ,10$$

Where $f_{\mathrm{ij}}^{L}$ and $f_{\mathrm{ij}}^{U}$ are the lower and upper rating of scenario *i* with respect to criterion *j* and $w_{j}$ represents the weight of each criterion that has resulted from the AHP method.

(d) Computing the VIKOR values (Q): For each scenario a Q interval is determined by using formulas (8) and (9)
  (8) $$Q_{i}^{L}=\upsilon \frac{\left(S_{i}^{L}-S^{*}\right)}{\left(S^{-}-S^{*}\right)}+\upsilon \frac{\left(R_{i}^{L}-R^{*}\right)}{\left(R^{-}-R^{*}\right)}\;i=1,\ldots ,10$$
  (9) $$Q_{i}^{U}=\upsilon \frac{\left(S_{i}^{U}-S^{*}\right)}{\left(S^{-}-S^{*}\right)}+\upsilon \frac{\left(R_{i}^{U}-R^{*}\right)}{\left(R^{-}-R^{*}\right)}\;i=1,\ldots ,10$$

Where υ is the weight of the strategy of the majority of criteria or the maximum group utility. Without loss of generality, it takes the value 0.5 and

$$S^{*}=\min_{i}S_{i}^{L},\;S^{-}=\max_{i}S_{i}^{U},\;R^{*}=\min_{i}R_{i}^{L},\;R^{-}=\min_{i}R_{i}^{L}$$

(e) Ranking the scenarios based on Q intervals: At the end, this method gives us the Q interval (relative distance from ideal solution) for each scenario. The best one is the scenario with the minimum Q; the Qs represent intervals that should be compared with each other and ranked in ascending order.

## 5. Result analysis and discussion

The decision matrix is shown in Table 8. In this matrix the rows represent the criteria and the columns represent scenarios. For each criterion there is a lower bound and upper bound, which refer to the 95% CI. In this matrix, the time unit is the minute and the cost unit is €1000. This matrix is used as an input to the VIKOR method. This method uses the matrix and the weight of each criterion to ranks scenarios. The final ranking is shown in Table 9. Each scenario is ranked from 1 to 4. The best scenario is scenario 6, which assigns priority based on the severity of the patient. In this scenario, patients with an acute condition receive services faster and have priority in various queues (patient and specimen queues). Implementing this scenario will reduce waiting time of acute patients about 5% without considering any additional investments. These scenarios can be joined and implemented simultaneously, which may have a synergic effect on our criteria. But if ED managers want to implement scenarios one by one, they should perform scenarios based on their ranking. All of these actions are at the operational level of decision making and they affect the system immediately. The ED seems to require prompt decisions at the tactical or even strategic level, such as buying an MRI machine and increasing the capacity of inpatient wards where utilization has risen to nearly 1, which has resulted in a dramatic increase of LWOR and waiting times.

**Table 8.** Decision matrix.

			Scenarios				
Criteria			S1	S6	S7	S8	S10
Waiting time (minutes)	ESI1	L	13.1	13.1	11.8	11.5	13.3
		U	20.4	18.0	25.5	23.1	17.3
	ESI2	L	115.2	113.0	108.6	123.1	108.0
		U	167.5	164.5	163.8	147.0	154.3
	ESI3	L	970.4	970.1	933.4	972.4	908.5
		U	1026	1015	1058	1030	980.4
	ESI4	L	1778	1842	1749	1763	1863
		U	1932	1944	1961	1954	1957
Utilization	Acute	L	0.533	0.522	0.536	0.522	0.528
		U	0.566	0.566	0.570	0.567	0.567
	Beds	L	0.988	0.988	0.988	0.988	0.987
		U	0.991	0.991	0.991	0.991	0.991
	CT	L	0.591	0.590	0.589	0.590	0.592
		U	0.597	0.596	0.596	0.596	0.600
	MRI	L	1.000	1.000	1.000	1.000	1.000
		U	1.000	1.000	1.000	1.000	1.000
	CT radiologist	L	0.820	0.820	0.821	0.821	0.821
		U	0.823	0.822	0.822	0.822	0.823
	MRI radiologist	L	0.666	0.667	0.555	0.665	0.665
		U	0.668	0.670	0.556	0.668	0.668
	MRI reception	L	0.538	0.539	0.449	0.450	0.540
		U	0.546	0.547	0.454	0.457	0.548
	MRI type	L	0.667	0.667	0.554	0.665	0.665
		U	0.668	0.668	0.556	0.668	0.667
	Pathologist	L	0.851	0.850	0.841	0.848	0.636
		U	0.864	0.862	0.875	0.863	0.651
	Screening doctor	L	0.818	0.822	0.820	0.809	0.827
		U	0.873	0.873	0.879	0.875	0.877
	Sonography radiologist	L	0.748	0.745	0.745	0.742	0.742
		U	0.752	0.752	0.756	0.753	0.759
	Triage	L	0.786	0.782	0.783	0.776	0.785
		U	0.839	0.839	0.845	0.838	0.842
LWOR		L	2293	2210	2307	2264	2266
		U	2452	2452	2452	2454	2445
Cost			0	0	15	3	8

ESI: Emergency Severity Index; CT: computed tomography; MRI: magnetic resonance imaging; LWOR: leave with own responsibility.

**Table 9.** Rankings.

Ranking	Scenarios	Q^L^	Q^U^
1	6	0	0.887771
2	7	0.07302	0.924954
3	10	0.261118	0.995326
4	8	0.118524	0.885526

This algorithm has some advantages. First, it is an interactive algorithm that takes the manager's idea into account. Managers who have a great insight into the ED situation lend a hand in determining the weight of each criterion and designing scenarios. Second, it considers uncertain conditions by regarding 95% CI data and consequently it reduces deviation from reality. Third, as this simulation is at the operational level of decision making and circumstances will alter continuously, it can tolerate changing and revising weights, scenarios or even the simulation model.

## 6. Future research directions

Several proposals could be outlined for future research directions. These are as following:

- In the above simulation model, historical data in HIS was used. As discrete event simulation is usually used for operating level of decision making, using real-time simulation is more operational with lower deviation from reality.
- The ED interacts widely with other parts of the hospital. In order to model the system and interactions of the ED, agent-based simulation can be used considering each part as an agent.
- In this research quantitative criteria have been considered. There are also some qualitative criteria such as staff satisfaction that could account for ED performance improvement. By using a fuzzy approach, fuzzy numbers are useful tools for converting these criteria to numbers. Fuzzy MADM methods could be used to solve this kind of problem.

## 7. Summary

This paper is about the performance of an ED of a government hospital in Iran. This ED faces serious challenges such as congestion, unbalanced utilization and long waiting times. These problems result in patient and ED staff dissatisfaction. This paper presents a method by which the performance of the ED could improve by using DES, DEA and MADM. A conceptual model that presents patient flow in the ED was used to construct a simulation model that reflects the behavior in the real system. Input data for the simulation was provided through HIS and interviews with ED staff, and the data were input into Input Analyzer and consequently the probability distribution of patient arrival and service times were computed. In our simulation model, the processes of medical tests such as laboratory and pathology testing, CT scans, MRI, and ultrasonography were detailed and virtual queues of patients' specimens were considered separately from patient queues. Patient arrival in the simulation model was considered to be non-homogenous in terms of time of day and day of the week. Before utilizing the simulation model, it was verified and validated.

Bottlenecks were identified based on simulation reports that presented the current ED situation. To resolve bottlenecks various scenarios were designed by considering conflicting criteria. These criteria were: waiting time, utilization, LWOR and cost. The Delphi method was used to specify a rational utilization rate for staff and equipment and the AHP method was used to specify the weight of each criterion. DEA was used in order to find efficient scenarios, and the extended VIKOR method was applied to rank the scenarios based on criteria derived from interval data, which was concluded from 95% CIs.

Scenario 6, which assigns priority based on the severity of the patient's condition, is the best scenario. In this scenario patients with acute conditions receive services faster and have priority in various queues (patient and specimen queues). Implementing this scenario will reduce waiting time for acute patients about 5% without considering any additional investments. The ED manager should apply a group of these scenarios that would considerably improve ED performance. But in this study we prepared a ranking of scenarios that gives decision makers an opportunity to compare and contrast scenarios and apply them one by one; then they can mix them and apply them simultaneously.
